# Cancer Immunotherapy: Priming the Host Immune Response with Live Attenuated *Salmonella enterica*

**DOI:** 10.1155/2018/2984247

**Published:** 2018-09-13

**Authors:** Marco Antonio Hernández-Luna, Rosendo Luria-Pérez

**Affiliations:** ^1^Department of Medicine and Nutrition, University of Guanajuato, Campus Leon, Leon, GTO, Mexico; ^2^Unit of Investigative Research on Oncological Diseases, Children's Hospital of Mexico Federico Gomez, Mexico City, Mexico

## Abstract

In recent years, cancer immunotherapy has undergone great advances because of our understanding of the immune response and the mechanisms through which tumor cells evade it. A century after the first immunotherapy attempt based on bacterial products described by William Coley, the use of live attenuated bacterial vectors has become a promising alternative in the fight against cancer. This review describes the role of live attenuated *Salmonella enterica* as an oncolytic and immunotherapeutic agent, due to its high affinity for tumor tissue and its ability to activate innate and adaptive antitumor immune response. Furthermore, its potential use as delivery system of tumor antigens and immunomodulatory molecules that induce tumor regression is also reviewed.

## 1. Introduction

Cancer is among the first causes of death in millions of individuals throughout the world [1]. The development of adverse effects and resistance to chemotherapy and radiotherapy, as well as the difficulty inherent to the elimination of metastatic cells, are some of the elements that underscore the need to search for better treatment alternatives with greater selectivity and effectiveness against tumor cells. Recent studies have documented the crucial role of the immune response in the elimination of tumors [2]; this fact has allowed to propose immunotherapy as an encouraging alternative in cancer treatment [3], by potentiating the host immune response activation or by acting in synergy with conventional treatments. In this context, the concept of using bacteria as agents against cancer described over a century ago [4] recently has generated great interest, as a result of the development of live attenuated bacterial vectors safe for human use, such as *Salmonella enterica*. This bacterium has proven usefulness in antitumoral therapy, by inducing innate and adaptive immune response in preclinical and clinical assays, which has led the tumor elimination without secondary effects [5], making *Salmonella enterica* a great candidate to cancer immunotherapy.

### 1.1. Bacteria in Antitumor Immunotherapy

The association of bacteria and antitumor activity was described in 1813, with observations of Vautier on tumor regression in patients with gangrene after *Clostridium perfringens* infection [6]. Subsequent studies by Coley, documented since 1890, demonstrated that “Coley's toxin,” constituted by *Streptococcus pyogenes* and *Serratia marcescens*, could immunotherapeutically treat patients with sarcomas, lymphomas, myelomas, and melanomas [4, 7]. Research initiated by Holmgren in 1935 [8], on the antitumor activity of the attenuated strain of *Mycobacterium bovis*, the Calmette-Guérin Bacillus (BCG), culminated in this approval of this strain in 1976, for intravesical application in patients with bladder superficial transitional cell carcinoma [9], a treatment modality that is currently still in use.

To date, the immunotherapeutic antitumor effect of bacteria has been proven in the genus *Bifidobacterium*, *Clostridium*, *Listeria*, *Escherichia*, and *Salmonella* [10, 11]. Among all these bacteria, *Salmonella enterica* serovar Typhi (*Salmonella* Typhi) and *Salmonella enterica* serovar Typhimurium (*Salmonella* Typhimurium) have been the most studied bacterial vectors in cancer treatment [12]. Some of the characteristics that make these vectors more suitable as antitumor immunotherapy are their property as facultative anaerobe bacteria [10]; their ability to colonize the tumor [13, 14], including metastasis [15]; and their affinity for professional antigen-presenting cells [16, 17], a characteristic associated with the induction or activation mechanisms of the innate immune response [18, 19] and the adaptive antitumor immune response [20, 21]. Furthermore, safe *Salmonella enterica* vaccine strains, such as Ty21a, are available in the market for human use.

### 1.2. The Selectivity of *Salmonella enterica* for Tumor Tissue

*Salmonella enterica*, a gram-negative bacterium, is highly selective for the tumor environment. However, the mechanisms mediating this characteristic need to be completely elucidated [14]. It has been well documented that tumor microenvironment, characterized by (a) hypoxia, oxygen concentrations ≤ 10 mmHg [10]; (b) the acidity conditioned by lactic acid, resulting from anaerobic metabolism because of decreased oxygen [22]; and (c) necrosis, resulting from tumor cell death due to lack of nutrients and uncontrolled growth [12], can contribute to bacterial proliferation in the tumor tissue. Likewise, some authors have suggested that *Salmonella enterica* migrates to the tumor tissue attracted by cell components that act as chemotactic agents, such as amino acids and carbohydrates [23, 24]. Recent studies have described the ability of *Salmonella* Typhimurium to sense the concentrations of ethanolamine, a part of membrane lipids, and hence colonize the gastrointestinal tract [25]. Interestingly, abnormal ethanolamine and other lipid levels of the cell membrane have been detected in different types of neoplasia [26], and they may be acting as chemoattractants of *Salmonella enterica* to the tumor [27].

On the other hand, there is controversial data on the role played by certain *Salmonella enterica* proteins involved in their ability to colonize tumor tissue, particularly the two-component system CheA/CheY; some authors mentioned that the presence of this system is indispensable for effective distribution and bacterial recruitment into tumor tissue [23, 24, 28]; its absence leads to decreased tumor colonization due to lower bacterial motility [29]. However, other studies have reported that the lack of CheY protein, as well as other bacterial components involved in motility such as the flagellar components fliA, fliC, and flgE, does not compromise *Samonella enterica* colonization of tumor tissue [27, 30, 31].

In spite of the discrepancies between the mechanisms used by *Salmonella enterica* to colonize the tumor, once *Salmonella enterica* reaches the tumor, its permanence in the tissue is associated to low macrophage and neutrophil activity due to the hypoxia within the tumor [32] and to the suppression of the immune response mediated by cytokines such as TGF-*β* [19] and the difficult access to the tumor microenvironment of preexisting anti-*Salmonella* antibodies and complement cascade factors due to the irregular growth of blood vessels in the tumor microenvironment [33]. In great measure, these mechanisms promote the antitumor immunotherapeutic activity of *Salmonella enterica* on different types of solid and semisolid tumors.

### 1.3. Intrinsic Oncolytic Activity of *Salmonella enterica*

**Figure 1:**
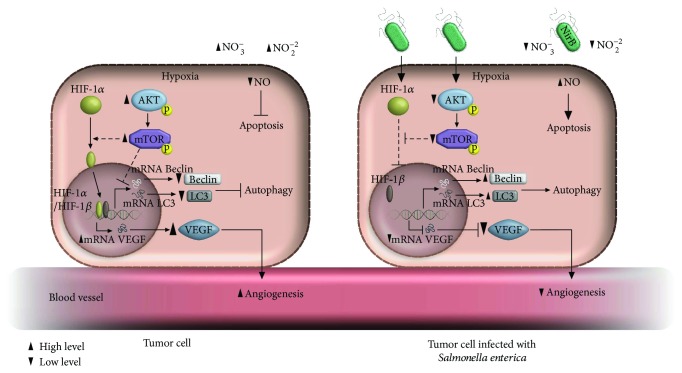
Oncolytic activity of *Salmonella enterica*. Once *Salmonella* reaches the tumor microenvironment, it promotes tumor cell elimination through several mechanisms: (A) inhibits tumor angiogenesis mediated by suppressing HIF-1*α* transcription factor of VEGF; (B) decreases AKT and mTOR phosphorylation, avoiding possible activation of HIF-1*α*, thus increases Beclin and LC3, two proteins required for autophagy; (C) degradation of nitrites and nitrates by the enzyme nitrite reductase (NirB) of *Salmonella enterica*, generates nitric oxide (NO) an apoptotic agent.

Preclinical and clinical studies have demonstrated the intrinsic antitumor capacity of *Salmonella enterica* (Table 1). This antitumor activity is partly explained by oncolytic mechanisms that are activated because of bacterial incorporation into the tumor microenvironment. Some of these mechanisms (Figure 1) are (1) competition for tumor cell nutrients [12]; (2) release of antitumor bacterial components due to lysis of the bacteria adhered to the tumor cell [34], such as *Salmonella enterica* nitrate reductase that metabolizes nitrates and nitrites [35], products of the hypoxic tumor environment [36] into nitric oxide (NO) [18], which has the ability to induce tumor cell apoptosis [37]; (3) decreased angiogenesis due to inhibition of the transcription factor HIF-1*α* and VEGF [38]; (4) activation of autophagy due to decreased phosphorylation of the proteins AKT and mTOR and increasing proteins as Beclin-1 and LC3 (microtubule-associated protein 1A/1B-light chain 3) [39, 40]; and (5) increased amounts of calreticulin [41], a protein associated to immunogenic cell death that is currently being evaluated as a possible therapeutic alternative in cancer [42].

Although the antitumor mechanisms of *Salmonella enterica* are not known in detail, several studies have documented this intrinsic activity in different tumor models (Table 1). In 1995, Eisenstein et al. showed that administration of the attenuated strain of S*almonella* Typhimurium SL3235 (mutant in the synthesis of aromatic amino acids (*aroA*)) inhibited the growth and decreased the size of the tumor mass in a plasmacytoma murine model [43]. Subsequent studies reported that attenuated *Salmonella enterica* strains not only decreased the size of the tumor but also delayed the development of metastases and increased survival in various murine cancer models, including melanoma [44], colon carcinoma [30, 45], prostate cancer [46], metastatic T-cell lymphoma [47], and B-cell lymphoma [48]. Similar results were obtained in xenograft mouse models of breast cancer [49] and prostate cancer [50, 51]. In these models, the auxotrophic strains of *Salmonella* Typhimurium, A1 strain (deficient in leucine and arginine synthesis) and the A1-R strain (deficient in leucine and arginine synthesis, with greater capacity to eliminate tumor cells), maintained their antitumor activity and did not cause toxic effects in the host due to its greater affinity for tumor tissue [49]. The A1-R strain also inhibited bone metastases from breast cancer [52, 53] and the metastases from osteosarcoma [54], pancreatic cancer [55, 56], and dorsal spinal cord gliomas [57].

Additionally, the antitumor efficacy of the A1-R *Salmonella enterica* strain has been evaluated *in vivo* in patient-derived orthotopic xenograft (PDOX) murine models [58, 59]. In these models, a fragment of a patient's tumor is surgically grafted into athymic naked mice (nu/nu) and once the mouse develops the tumor, it is treated with the live attenuated bacterium. Metastatic colon cancer PDOX models have also been developed [58], as well as osteosarcoma [60–62], melanoma [63–67], follicular dendritic cell sarcoma [68], and soft tissue sarcoma [69, 70]. These models have shown that the intraperitoneal, intravenous, or intra-arterial administration of *Salmonella enterica* A1-R colonizes and decreases the size of the tumor. PDOX models have also revealed that *Salmonella enterica* A1-R can eliminate tumor cells that are resistant to chemotherapeutic agents such as cisplatin [60, 61, 67], doxorubicin [68, 69], and temozolomide [64]. Likewise, *Salmonella enterica* A1-R eliminated the tumors in PDOX models resistant to kinase inhibitors such as sorafenib [62] and vemurafenib [65]. These studies show the potential clinical usefulness of *Salmonella enterica* A1-R in antitumor therapy.

Some clinical trials have reported the use of live attenuated *Salmonella enterica* strains in the treatment of cancer. A phase I clinical study using the VNP20009 strain of *Salmonella* Typhimurium, with mutations in the *msbB* genes (affecting the formation of lipid A, decreasing the toxicity associated to the lipopolysaccharide) and *purI* genes (turning it dependent on an external adenine source), included 24 patients with metastatic melanoma and 1 patient with metastatic renal cell carcinoma; in the study, patients that received an intravenous dose of the VNP20009 strain did not develop adverse reactions to the *Salmonella enterica* infection, but bacterial colonization was moderate and the antitumor effect was not significant [71]. Further studies have shown that the antitumor activity failure could have resulted from low bacterial colonization of the tumor tissue, since the VNP20009 strain has a polymorphism in the CheY gene [29] and a mutation in the *msbB* gene [72], associated with low strain motility. Indeed, the presence of previous antibodies against *Salmonella enterica* could also have been a factor compromising antitumor activity [73].

### 1.4. Activation of the Antitumor Innate Response by *Salmonella enterica*

**Figure 2:**
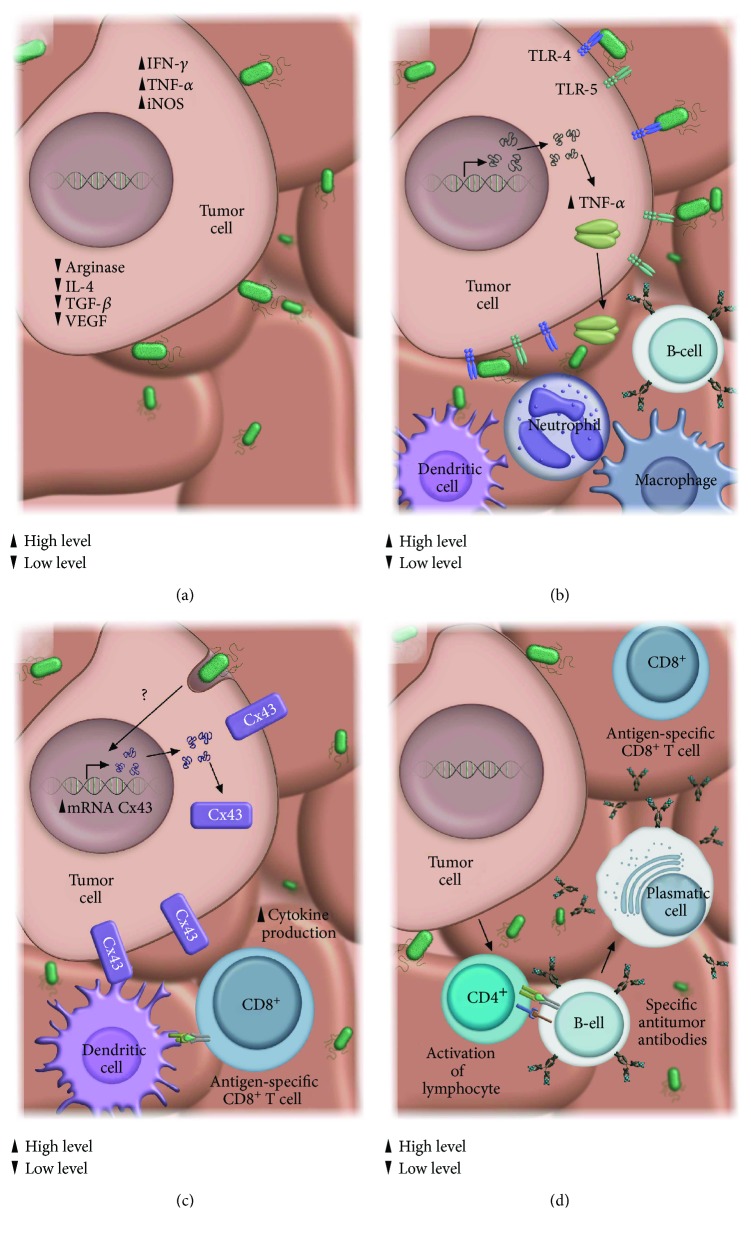
Activation of innate and adaptive immune response in the tumor microenviroment by *Salmonella enterica*. Once *Salmonella* colonizes tumor tissue, it induces an antitumor innate and adaptive immune response through several mechanisms: (a) promotes proinflammatory cytokines (IFN-*γ* and TNF-*α*), while decreases both anti-inflammatory (TGF-*β*, IL-4) and angiogenic factors (VEGF) associated with tumor growth progression; (b) interactions between bacterial components (LPS and flagellin) and tumor cell receptors as TLR4 or TLR5, respectively, induce cytokine secretions that promotes the recruitment of neutrophils, macrophages, T lymphocytes, B lymphocytes, and dendritic cells to the tumor microenvironment; (c) *Salmonella* colonization induces the expression of connexin 43; this molecule plays a major role in the cross-presentation of tumor antigens by DCs to CD8^+^ T-cells; (d) the presence of antitumor CD4^+^ T-cell induce the activation and differentiation of B lymphocytes into plasma cells, producing specific antitumor antibodies.

In the tumor microenvironment, immunosurveillance evasion mechanisms prevent the eradication of tumor cells [2] and represent a barrier that *Salmonella enterica* must overcome when used as an immunotherapeutic agent. The first studies describing the antitumor immunotherapeutic properties of *Salmonella enterica* were conducted by Kurashige et al., using minicells (vesicles with no genomic DNA) obtained from *Salmonella* Typhimurium, and evaluated in two different murine models (sarcoma [74] and T-cell lymphoma [75]); they observed that the administration of these minicells restored macrophage activity in the tumor microenvironment, promoting tumor elimination. Recent studies have reported that some of the mechanisms that could use the bacterium to eliminate the tumor cells once it is in the tumor microenvironment involved enhance the expression of soluble mediators such as inducible nitric oxide synthase (iNOS) and interferon *γ* (IFN-*γ*) and also inhibit the expression of immunosuppressive factors such as arginase-1, interleukin-4 (IL-4), transforming growth factor-*β* (TGF-*β*), and vascular endothelial growth factor (VEGF) [19] (Figure 2(a)). In addition, *Salmonella enterica* also can decrease the activity of myeloid-derived suppressor cells (MDSCs) within the tumor microenvironment [76] and promotes the recruitment of NK cells [77], neutrophils [18], macrophages [19], and T [21] and B lymphocytes [20] into the tumor microenvironment and spleen [41].

Other studies have documented the ability of *Salmonella enterica* to suppress tumor growth inducing inflammasome [78], by activation of interleukin-1*β* (IL-1*β*) and TNF-*α* [79]. Likewise, *Salmonella enterica* also increases the levels of proinflammatory cytokines while decreasing the levels of antiinflammatory cytokines in the tumor microenvironment [80], and this modulation of cytokines may result from the activation of Toll-like receptors (TLRs) in the tumor tissue.

### 1.5. Antitumor Response of *Salmonella enterica* by TLR Activation

Activation of the host innate immune response via TLRs is one of the therapeutic strategies against cancer that has begun to be evaluated [81]. Studies in which TLR4 is activated by *Salmonella choleraesuis* revealed less tumor growth in a melanoma murine model, and this decrease was associated to the recruitment of innate immune response cells such as neutrophils and macrophages [82]. On the other hand, the activation of TLR5 by the flagellin of *Salmonella* Typhimurium fused with peptide P10 of the gp43 protein of *Paracoccidioides brasiliensis* eliminated the development of metastases in the melanoma murine model [83], and the use of a TLR5 agonist displayed antitumor effects in a murine lymphoma model while also promoting the activation of CD8^+^ lymphocytes and NK cells [84]. Although, these studies showed a possible role of TLR5 in cancer treatment, a recent study conducted by J.H. Zheng et al. that evaluate the antitumor effect of TLR4 and TLR5 by *Salmonella enterica*, using knockout (KO) mice for these receptors, shown differences in the antitumor capacity between both receptors. In this study, mice bearing colon cancer or melanoma implant were treated with a *Salmonella* Typhimurium that expresses flagellin B (FlaB) of *Vibrio vulnificus*. The results showed that TLR4 KO mice had exacerbated tumor development, similar to those seen in mice that were not treated with *Salmonella* Typhimurium; on the other hand, TLR5 KO mice showed a partial decrease in tumor growth. Total tumor reversal was observed, as expected, in wild-type mice that had also been implanted with tumor cells and treated with the same *Salmonella* Typhimurium expressing FlaB [85]. This data shows that the main effect of this bacterium was mediated by TLR4 activation, while TLR5 plays a less decisive role in tumor suppression. These observations are consistent with results from other studies, in which the administration of *Salmonella* Typhimurium flagellin in a breast cancer murine model had no significant antitumor effect if the flagellin is administered after tumor implantation, but interestingly, the simultaneous administration of flagellin and tumor cells promoted faster tumor development in the mice [86]. Studies conducted in a multiple myeloma cell line support the controversial data on the role of TLR5, since its activation promoted the proliferation and tumor cell survival [87].

### 1.6. Tumor Cell Immune Response to *Salmonella* Infection

The recruitment of immune response cells in the tumor microenvironment, including NK cells (natural killer), neutrophils [18], macrophages [19], T lymphocytes [21], and B lymphocytes [20], has been described as one of the main mechanisms through which *Salmonella enterica* is able to eliminate tumor cells. Although the mechanisms involved in the initial recruitment of these cells, after intratumoral administration of *Salmonella enterica*, remain under study [88], this process could begin by recognizing bacterial LPS via TLR4, leading to increased TNF-*α* levels [89], which provoke hemorrhage from the blood vessels in the tumor, thus promoting infiltration of the immune response cells, which will initiate the tumor elimination process [90] (Figure 2(b)). On the other hand, B and T lymphocyte responses resulting from the administration of S*almonella enterica* also play a significant role in the antitumor effect. In this context, the depletion of B lymphocyte promotes preferential colonization of *Salmonella enterica* in tumor tissue and also in organs such as the spleen and liver and increases the permanence of bacterium in the blood [20]. However, these colonization differences were not observed after depletion of CD4^+^ and/or CD8^+^ cells, but a decrease in the recruitment of neutrophils and macrophages in tumor tissue was observed [21].

Further, the antitumor effect of *Salmonella enterica* also includes the dendritic cells (DCs). A study by Shilling et al. [91] showed that *in vitro* activation of purified murine DCs with cytoplasmic *Salmonella* Typhimurium fractions and tumor-derived heat shock proteins prevented tumor formation after DCs activated were reinoculated in mice; however, DCs that were only activated with the cytoplasmic fractions of bacterium or with the tumor-derived heat shock proteins did not prevent tumor growth. Additionally, they showed that DCs activated were preferentially localized in the tumor, followed by lymph nodes and in a lower proportion, in the liver, lung, and spleen. Other studies have documented that *Salmonella enterica* also favors the cross-presentation of tumor antigens by DCs and induces CD8^+^ lymphocyte activation capable of recognizing tumor cells [80]; this could be associated with the generation of a protective effect that prevents tumor relapse [89]. Nevertheless, the last date must be confirmed, since a study conducted in a murine melanoma model revealed a specific response against the tumor mediated by CD8^+^ lymphocytes but did not induce an immunologic memory [92].

Moreover, several studies have documented that *Salmonella enterica* triggers tumor regression by reverting its immune tolerance, through two possible mechanisms: (1) by decreasing the amount of T-regulatory lymphocytes CD4^+^ CD25^+^ (Treg) in tumor tissue by the effect of LPS and the Braun lipoprotein (Lpp) of *Salmonella enterica*, because mutations in *msbB* gene and *IppA e IppB* genes (Lpp) do not decrease the number of Treg lymphocytes in the tumor [93], and (2) by decreasing the levels of the enzyme indoleamine 2,3-dioxygenase-1 (IDO1) [40], an enzyme of tryptophan metabolism associated to the development of immune tolerance in T lymphocytes [94, 95], preventing the formation of kynurenine and promoting the proliferation of T lymphocytes capable of recognizing and eliminating the tumor.

### 1.7. Induction of the Antitumor Adaptive Immune Response by *Salmonella enterica*

The adaptive immune response also plays an important role in the antitumor activity induced by *Salmonella enterica*, because the response against *Salmonella enterica* antigens has been considered as a possible mechanism for tumor elimination [96, 97]. Although the mechanism is not completely understood, it has been proposed that once the bacterium reaches the tumor microenvironment, infected tumor cells are capable to process and present *Salmonella enterica* antigens to cytotoxic T lymphocytes that eliminate infected cells; this process has been observed in solid tumors and their metastases [50, 98], as well as, nonsolid tumors [77]. Other mechanism that could use *Salmonella enterica* to activate immune response is enhancing the expression of connexin 43 (Cx43) [99], a protein associated to B and T lymphocyte activation [100], and promotes the cross-presentation of tumor cell antigens by dendritic cells [101], through the formation of gap junctions that allow the passage of preprocessed tumor cell peptides into the dendritic cell for adequate presentation by MHC class I [99], thus favoring the CD8^+^ T antitumor lymphocytes (Figure 2(c)). Other studies have described the ability of *Salmonella enterica* to induce T lymphocyte proliferation [40] and increase the levels of antitumor proinflammatory cytokines [80]. For instance, in a murine B-cell lymphoma model, the administration of *Salmonella* Typhimurium induced a local and systemic adaptive antitumor immune response, characterized by the recruitment of CD8^+^ and CD4^+^ lymphocytes into the tumor [77]; likewise, it was observed that the lymphocytes obtained from the spleen of these mice secreted proinflammatory cytokines, such as IFN-*γ* and IL-12, in response to the specific stimulus by tumor cells, and the analysis of the humoral response revealed the presence of specific antibodies against tumor cells, which contribute to tumor eradication (Figure 2(d)).

### 1.8. *Salmonella enterica* as Delivery System of Tumor-Associated Antigens or Tumor-Specific Antigens for Cancer Therapy

Although many studies using murine models have shown the oncolytic activity of *Salmonella enterica*, in a clinical trial, it was observed that bacterium was not sufficient to eliminate the tumor [71]. In order to improve the antitumor potency of this bacterial vector, *Salmonella enterica* has been used as a delivery system of tumor-associated antigen (TAA) or tumor-specific antigen (TSA) [102] (Table 2), proteins expressed on tumor cells that promote transformation and tumorigenesis. The expression of these antigens on *Salmonella enterica* has the purpose of inducing or potentiating the specific immune response against the tumor, considering the great tropism of *Salmonella enterica* for professional antigen-presenting cells [103]. With this purpose, the expression and releasing of TAA/TSA through type 1 (T1SS) and type 3 (T3SS) secretion systems of *Salmonella enterica* have been documented. For instance, mice immunization with a *Salmonella* Typhimurium strain that released prostate-specific antigen (PSA) via the HlyA (T1SS) system activated an immune response mediated by CD8^+^ T lymphocytes, which inhibited tumor development [104]. Likewise, immunization of a murine pulmonary adenoma model with *Salmonella* Typhimurium overexpressing the C-Raf antigen (a molecule with a central role in carcinogenesis) induced antibodies against this protein, generating an antigen-specific T-cell response and inhibiting tumor growth [105]. Moreover, the release of peptide of the *Listeria monocytogenes* p60 protein, simulating the presence of a tumor antigen via T3SS of *Salmonella* Typhimurium in a fibrosarcoma murine model, demonstrated that 80% of mice immunized were protected after a fibrosarcoma tumor cell challenge that expressed the p60 peptide; this effect would be associated to the presence of CD8^+^ T lymphocytes specific against this peptide [106, 107]. Similar results have been observed after oral immunization with an attenuated strain of *Salmonella enterica* that releases the tumor antigen NY-ESO-1 (a protein in germ cells that is overexpressed in cancer of the lung, melanoma, esophagus, ovary, bladder, and prostate) via T3SS [108]. Likewise, orogastric immunization with *Salmonella* Typhimurium, which translocates the immunogenic epitope of the murine vascular endothelial growth factor receptor 2 (VEGFR-2) via T3SS, induced an antigen-specific immune response by CD8^+^ T lymphocytes, in a murine melanoma model, and decreased metastases up to 60% in the immunized mice [109]. Other study showed that the release of the recombinant protein E7/SipB (E7 protein of the human papillomavirus, type 16/SipB, protein of T3SS) in a cervical cancer murine model inhibited tumor growth to 45% and promoted mouse survival up to 70% [110].

On the other hand, the ability of *Salmonella enterica* to transfer nucleic acids into a eukaryote cell (bactofection) has also been evaluated in the generation of a tumor antigen-specific immune response. In this context, the bactofection of the *L1 HPV16* gene, which encodes the capsid protein of the type 16 human papillomavirus, in cervical cancer murine model with a strain of *Salmonella enterica*, led to tumor regression and increased the survival of mice [111]. Likewise, in a breast cancer murine model in which *Salmonella enterica* performed the bactofection of the gene encoding the protein MTDH/AEG1-1, an oncogene associated to angiogenesis that is overexpressed in 40% of breast cancer patients, tumor regression was also observed as well as increased survival of the mice [112].

*Salmonella enterica* was recently used to transport 4-1IBBL molecules, a member of the TNF family, and CEACAM 6 molecules, a cellular adhesion molecule, in a rat colon cancer model; the immunization with *Salmonella enterica* carrying those antigens avoided tumor progression, decreased the numbers of Treg cells, promoted a Th1 response, and increased the numbers of CD45RO^+^ memory T-cells [113].

### 1.9. *Salmonella enterica* as Delivery System of Immunomodulating and Apoptosis-Inducing Proteins for Cancer Therapy

The use of *Salmonella enterica* as a tumor antigen carrier in CD4^+^ and CD8^+^ T lymphocyte activation is limited to those immunogenic tumors expressing associated or specific tumor antigens [13]. An alternative to this inconvenience is the use of *Salmonella enterica* as a delivery system of molecules that modulate the immune response of the host, facilitating the elimination of tumor. *Salmonella* Typhimurium has indeed been used to transport immunomodulating proteins such as LIGHT [114], interleukin-18 [115], and the chemokine CCL21 [33], in breast cancer and colon cancer murine models; in all cases, regression of the primary tumor was observed as well as of its pulmonary metastases, where the antitumor activity was associated to the recruitment of DCs, macrophages, neutrophils, NK cells, and lymphocytes. Other studies conducted with *Salmonella* Typhimurium expressing human interleukin-2 prevented the formation of pulmonary metastases in an osteosarcoma murine model in which NK cells were possibly responsible for the tumor regression [116, 117]. Also, the use of *Salmonella enterica* in the bactofection of plasmids encoding interleukin-4 or interleukin-18 induced a systemic increase in IFN-*γ* and was efficient in delaying tumor growth and prolonging survival in a melanoma murine model [118] (Table 2).

Additionally, aside from the expression of immune-modulating molecules, *Salmonella enterica* has also been used to express and/or secrete molecules that induce tumor cell death by apoptosis, such as Fas ligand [119], TNF-*α* [120], or TRAIL (tumor necrosis factor-related apoptosis-inducing ligand) [121], in murine models of colon cancer, melanoma, or gastric cancer, respectively; in all cases, significant tumor regression was observed as well as increased mouse survival.

### 1.10. Is *Salmonella* Typhi the Most Appropriate Immunotherapeutic Agent?

Most studies documenting the role of *Salmonella enterica* as an antitumor immunotherapeutic agent have been conducted with the attenuated *Salmonella* Typhimurium strain, a species that in case of pathogenicity would only cause a mild infection in humans, since it preferentially infects mice [96]. However, the modest antitumor activity induced by this species in clinical trials [71] raises the possibility of using *Salmonella* Typhi vaccine strains (whose natural host is the human), such as the Ty21a strain [122] and the CVD915 strain [123], for antitumor immunotherapeutic purposes in humans. There are a few studies evaluating the ability of these *Salmonella* Typhi vaccine strains to act as immunotherapeutic agents. The CVD915 strain was evaluated in a breast cancer murine model, and it delayed tumor growth in association with CD8^+^ and B220^+^ lymphocyte activation, but not CD4^+^ cells. Another study revealed that the administration of this same strain decreased the amount of Treg lymphocytes in the tumor area [124]. Additionally, in a T-cell lymphoma murine model treated with the CVD915 strain, a decrease in metastasis toward lymph nodes was observed [47]. These results were consistent with those obtained in a breast cancer murine model, in which the administration of *Salmonella* Typhi CVD915 prevented metastasis development due to previous activation of B and T lymphocytes and DCs [125]. Also, studies using the Ty21a vaccine strain in a bladder cancer murine model led to tumor regression by CD8^+^ T lymphocyte activation and the expression of chemokines such as CXCL5, CXCL2, CCL8, and CCL5 [126]. These results have established the basis for the use of strains such as *Salmonella* Typhi Ty21a with antitumor purposes. Recently, a phase I clinical trial was conducted in patients with stage IV pancreatic cancer, which used *Salmonella* Typhi Ty21a for bactofection of a plasmid with the human VEGFR-2 sequence (overexpressed protein on endothelium of tumor microenvironment); its aim was to induce an antiangiogenic response and memory immune response against endothelial cells to eliminate tumor vascularization [127]. Preliminary results revealed that treatment with *Salmonella* Typhi Ty21a was well tolerated by patients and led to significant tumor regression [128]. This data significantly reinforces the use of *Salmonella* Typhi as an antitumor immunotherapeutic agent that can be used in a biosafety manner in the treatment of cancer.

## 2. Conclusion

Bacteria played a key role in the early stages of antitumor immunotherapy with the use of Coley's toxin [4], a therapeutic modality that was substituted by the advent of radiotherapy and chemotherapy. However, the use of the attenuated strain of *Mycobacterium bovis*, BCG, in the treatment of patients with superficial transitional cell bladder cancer is an active option to this day [9]. This review has described in detail the use of live attenuated *Salmonella enterica* as the immunotherapeutic bacterial vector par excellence, in cancer treatment. This bacterium fulfills all the characteristics required by a live attenuated bacterial vector to act as an immunotherapeutic agent [129]: (a) its biology must be fully known, including its facultative anaerobe property [96], which facilitates its selectivity for the tumor microenvironment and its intrinsic oncolytic activity; (b) for decades, it has been described as a bacterial vector with vaccine purposes due to its high affinity for professional antigen-presenting cells, favoring immunotherapeutic activity in the induction of the innate and adaptive antitumor immune responses [16, 17]; (c) there are biologically safe attenuated strains for immunotherapeutic use in humans [71, 127, 128]; and (d) its capacity as a delivery system of immunomodulating molecules [33, 115, 116] and TAA/TSA [53, 105, 109] has been proven to facilitate antitumoral immunotherapeutic activity.

Finally, based on the above mentioned studies, we can conclude that *Salmonella enterica* may be currently considered a live attenuated bacterial vector with great potential in the field of cancer immunotherapy.

### 2.1. Future Directions

Over a century and a half after the first reports on bacterial antitumor activity [7, 130], live attenuated *Salmonella enterica* has been consolidated as an ally in cancer therapy [5, 11, 12, 131]. However, although some clinical trials have been reported (Tables 1 and 2), their number should be increased with different malignant neoplasms using *Salmonella enterica* as an alternative antitumor therapy, including the possibility of using the bacterium in combination with chemotherapeutic agents [132]. Research efforts also should be focused on developing better and biosafe live attenuated strains, optimizing the production and transport mechanisms of antitumor molecules into the cell or cellular microenvironment, and improving the bacterium's selectivity for the cell or tumor tissue. Regarding the development of live attenuated biosafe strains, aside from using *Salmonella Typhimurium* strain VNP20009 that has been proven to be well tolerated by patients with metastatic melanoma, metastatic renal carcinoma, head and neck carcinoma, and esophageal adenocarcinoma [71, 133, 134], recent studies have focused on the use of *Salmonella Typhi* strain Ty21a, a biosafe strain approved for human use as a vaccine [127]. In this context, some options that should be evaluated in antitumor therapy in its clinical phases are the *Salmonella* Typhi CVD908, CVD908-htrA, and Ty800 strains that have also been proven to be safe in vaccine clinical trials [17, 135]. Several efforts have been described to improve the production and transport of antitumor molecules into the cell or cellular microenvironment, as reflected in recent studies describing a *Salmonella enterica* with a self-limited lifecycle controlled by a lysis circuit that allows the bacterium to release in an oscillatory manner the cytotoxic antitumor molecule [136]; an interesting strategy that could be evaluated is to use the bacterial secretion systems, such as type V or autotransporter, a mechanism present in *Salmonella enterica* that could release antitumor heterologous molecules coupled to peptides that destabilize cell membranes in order to reach the target in the tumor cell [11, 137]. Increasing the bacterium's selectivity for the cell or tumor tissue will help decrease the secondary effects inherent to the bacterium's intrinsic toxicity; some improvements have been developed by coupling single-domain antibodies to the surface of *Salmonella Typhimurium* SL3262 in order to increase the bacterium's specificity for the tumor microenvironment [138]. Another alternative that could increase this selectivity and that should be evaluated is the use of synthetic adhesins fused to the variable domains of the antibody's heavy chain that once expressed in bacteria and have shown to be efficient in colonizing tumors expressing some antigen recognized by the synthetic adhesin [139].

Accordingly, obtaining the best live attenuated and biosafe strains, clinically tested, which induce minimal side effects and that still exert their antitumor effect, will allow to confirm that live attenuated *Salmonella enterica* is the vector par excellence in cancer immunotherapy.

## Figures and Tables

**Table 1:** Antitumoral intrinsic activity of *Salmonella enterica*.

Specie	Strain	Mutation	Tumor-bearing mice	Administration	Reference
*Salmonella* Typhimurium	S14028	*pur*: *ilv*; *arg*; *ura*; *aro*	Melanoma	Intraperitoneally	[14]
*Salmonella* Typhimurium	SL7207 SL1344	*aroA*, *hisG46*; *cheY*; *fliGHI*; *invG*; *phoP*; *sseD*; *ssrB*; *purA*	Colon cancer	Intravenously, intraperitoneally	[30]
*Salmonella* Typhimurium	SL3235	*aroA*	Plasmocytoma	Intraperitoneally	[43]
*Salmonella* Typhimurium	VNP20009	*purI*, *msbB*	Melanoma	Orally, intraperitoneally	[44]
*Salmonella* Typhimurium	Wild-type LT2	Δ*ppGpp hisD2550*, *rpoS*, *aroA*, *rfaH*, *thyA*	Prostate cancer	Intraperitoneally	[46]
*Salmonella* Typhimurium	14028 auxotrophy A1 and A1-R	*Leu*, *Arg*	Prostate cancer	Intratumorally, intravenously	[50, 51]
			Breast cancer	Intravenously	[49]
			Breast cancer bone metastasis	Intravenously	[53]
			Bone tumor and lung metastasis of osteosarcoma	Intravenously	[54]
			Pancreatic cancer	Intraperitoneally, Intratumorally	[55, 56]
			Spinal cord glioma	Intravenously	[57]
*Salmonella* Typhimurium	LVR01	*aroC*	B-cell lymphoma	Intratumorally	[48]
*Salmonella* Typhi	CVD915	*guaBA*	T-cell lymphoma	Intratumorally, subcutaneously	[47]

*PDOX Models*					
*Salmonella* Typhimurium	A1-R	*Leu*, *Arg*	Osteosarcoma metastasis	Intravenously	[60]
			Osteosarcoma	Intra-arterial	[61]
				Intratumorally	[62]
			Melanoma	Orally	[64]
				Intravenously	[65–67]
			Follicular dendritic cell sarcoma	Intraperitoneally	[68]
			Soft tissue sarcoma	Intratumorally	[69]
				Intravenously	[70]

*Clinical Trial Phase I*					
*Salmonella* Typhimurium	VNP20009	*purI*, *msbB*	Metastasic melanoma	Intravenously	[71]

**Table 2:** *Salmonella enterica* as delivery system of TAA/TSA and immunomodulatory molecules.

Species	Mutations	Heterologous molecule	Tumor-bearing mice	Antitumoral response	Reference
*Tumor-associated antigens or tumor-specific antigens (TAA/TSA)*					
*Salmonella* Typhimurium	*aroA*	Antigen PSA	Prostate cancer	Cytotoxic CD8^+^ T-cell	[104]
	*aroA*	L1HPV16	Cervical cancer	IFN-*γ* and TNF-*α* secretion	[111]
	*aroA*, *sptP*	Antigen p60_217–225_	Fibrosarcoma	Effector and memory CD8^+^ T-cells	[106, 107]
		VEGFR-2	Melanoma	Specific CD8 T-cell	[109]
	*aroA*, *hisG46*	C-Raf	Lung adenoma	Humoral and T-cell responses	[105]
	*aroA*, *aroD*	SipB160-HPV16E7	Cervical cancer	Humoral response	[110]
*Salmonella* Typhimurium	*phoP*, *phoQ*	NY-ESO-1	Sarcoma	NY-ESO-1-specific CD8^+^ T-cell response	[108]
*Salmonella* Typhimurium	*dam*, *aroA*	MTDH/AEG1-1	Breast cancer and metastasis	CD8^+^ T-cell response	[112]

*Immunomodulatory and apoptosis inductor molecules*					
*Salmonella* Typhimurium	*purl*, *msbB*	CCL21	Colon and breast cancer	CD4^+^ and CD8^+^ T-cell response	[33]
		LIGHT	Colon and breast cancer	Infiltration of inflammatory cells	[114]
		IL-18	Colon and breast cancer	CD4^+^ T-cell response and NK cells	[115]
		FasL	Colon and breast cancer	Accumulation of neutrophils	[119]
*Salmonella* Typhimurium	*cya*, *crp*, *asd*	IL-2	Osteosarcoma and metastasis	NK cells	[116, 117]
*Salmonella* Typhimurium	*aroA*	IL-4 and IL-18	Melanoma	Increase levels of IFN-*γ*	[118]
	*aroA*, *hisG46*	TRAIL	Gastric cancer	Increase levels of caspases 3 and 9	[121]
	*aroA*, *aroD*	TNF*α*	Melanoma	NK cells	[120]

*Clinical Trial Phase l*					
*Salmonella Typhi*	*galE*	VEGFR-2	Pancreatic cancer	T-cell response	[127, 128]

CCL21: chemokine (C-C motif) ligand 21; C-Raf1: serine-threonine kinases of the Raf family; FasL: Fas ligand; IL-2: interleukin-2; IL-4: interleukin-4; IL-18: interleukin-18; LIGHT: a member of TNF cytokine family; L1HPV16: capsid protein L1HPV16; MTDH: metadherin; NY-ESO-1: testis antigen; PSA: prostate-specific antigen; HPV16E7: human papillomavirus protein E7; TRAIL: TNF-related apoptosis-inducing ligand; TNF-*α*: tumor necrosis factor *α*; VEGFR-2: vascular endothelial growth factor receptor-2; NK: natural killer; IFN-*γ*: interferon *γ*.
